# Supplementary material for: Intestinal-Type Adenocarcinoma Is a Rare Histotype of Vulvar Neoplasm: Systematic Review of the Literature
Source: Cancers (Basel). 2025 Dec 14;17(24):3989. doi: 10.3390/cancers17243989 (PMC12731156; doi:10.3390/cancers17243989)
Supplement: Supplementary file 1 [file cancers-17-03989-s001.zip › cancers-3963932-supplementary.pdf]

cancers MDPI

Supplementary Material S1. Case of VAIt From Our Institution.

We reported our patient case following the Consensus-based Clinical Case Reporting (CARE) Guideline Development [51]. We collected de-identified patient data, including clinical history, physical examination findings, imaging studies, histological reports, and treatment details. The case was documented and followed prospectively through treatment and follow-up. Written informed consent was obtained from the patient for the publication of this case report and any accompanying images. Documentation is available upon request.

A 58 years old woman arrived at our Institution (Responsible Research Hospital of Campobasso [Italy]), in January 2025. She was diagnosed with a vulvar carcinoma in 2021 and treated in another hospital with exclusive chemotherapy and radiotherapy. In late 2024, the patient presented with a neoplastic lesion of the vulva associated with bilateral inguinal lymphadenopathy. A biopsy of the inguinal lymph nodes was performed in another Institution, reporting a histopathological diagnosis of "metastatic mucinous adenocarcinoma of vulvar origin". Upon presentation at our institution, a gynecological examination revealed an inguinal tumefaction and a verrucous neoplastic lesion extending from the vaginal wall to the distal lower third of the right hemivulva. An excisional biopsy of this lesion was performed, with a histopathologic diagnosis of "mucinous adenocarcinoma exhibiting intestinal differentiation with signet ring cell morphology". Immunohistochemical analysis demonstrated positivity to CK20 (weak to moderate), Ca19.9 (weak to moderate), CEA (strong), p16 (weak to moderate), and focally positive for Ca125; while negative for CK7, ER, PR, PAX8, and GCDFP15. Pelvic magnetic resonance imaging (MRI) demonstrated an ill-defined area of heterogeneity within the right inferolateral vaginal and vulvar walls, characterized by an altered signal intensity (maximum dimension of 30 mm antero-posteriorly and 19 mm latero-laterally) (Supplementary Figures S1 and S2). This region displayed heterogeneous T2 hyperintensity, heterogeneous restricted diffusion on diffusion-weighted imaging (DWI), and heterogeneous post-contrastographic enhancement, with an associated area of liquefactive necrosis (maximum diameter approximately 23 mm). Bilateral inguinal lymphadenopathies were noted: on the right (measuring approximately 19 mm in maximum diameter, exhibiting an irregularly restricted signal and partial necrosis) and on the left (ovoid morphology, with maximum diameter of 20 mm, also showing an irregularly restricted signal and partial necrosis). No significant lymphadenopathies were observed in the internal iliac nodal stations. No significant pelvic effusion was present. Total-body 18F-fluorodeoxyglucose positron emission tomography-computed tomography (18F-FDG PET-CT) scan confirmed the presence of a focal capture of radiotracer in correspondence of a tissue area with inhomogeneous density located in the vulvar area with greater extension on the right with SUV max 6.3 and in correspondence of lymph node formations located in the right common iliac area with SUV max 1.7, in the left obturator area with SUV max 3.7, in the left external iliac area with SUV max 1.8 and bilateral inguinal however, diagnostic imaging did not reveal evidence of secondary neoplastic foci in the upper abdominal cavity or systemic distant metastases. For the exclusion of a primary colorectal malignancy, the patient underwent esophagogastroduodenoscopy and colonoscopy, which revealed no evidence of suspicious heteroplastic lesions. The clinical case was reviewed at the multidisciplinary tumor board of our institution: the decision was to proceed with a debulking surgical procedure. In March 2025, the patient underwent laparoscopic radical hysterectomy type B2 according to the Querleu-Morrow classification, bilateral adnexectomy, systematic pelvic

lymphadenectomy (level I according to the Querleu-Morrow classification), radical vulvectomy with "en-bloc" total colpectomy, and bilateral inguinal lymph node dissection. Definitive histological examination reported metastasis of mucinous adenocarcinoma with intestinal mucinous differentiation, predominantly exhibiting signet ring cell morphology, in 9 of 9 left inguinal lymph nodes excised and in 11 of 11 pelvic lymph nodes. The vulva and vagina were sites of poorly differentiated mucinous adenocarcinoma exhibiting signet ring cell features (Supplementary Figure s3). Extensive LVSI was present and perineural infiltration was identified. Resection margins were free of disease. The patient was discharged after a 5-day postoperative hospital stay in good general condition and without complications. According to multidisciplinary Tumor Board Group's decision, the patient started an adjuvant chemotherapy regimen consisting of six cycles of carboplatin and paclitaxel. Initiation of adjuvant treatment occurred one-month after surgical intervention. To date the patient is free from relapses and late post-operative complications.

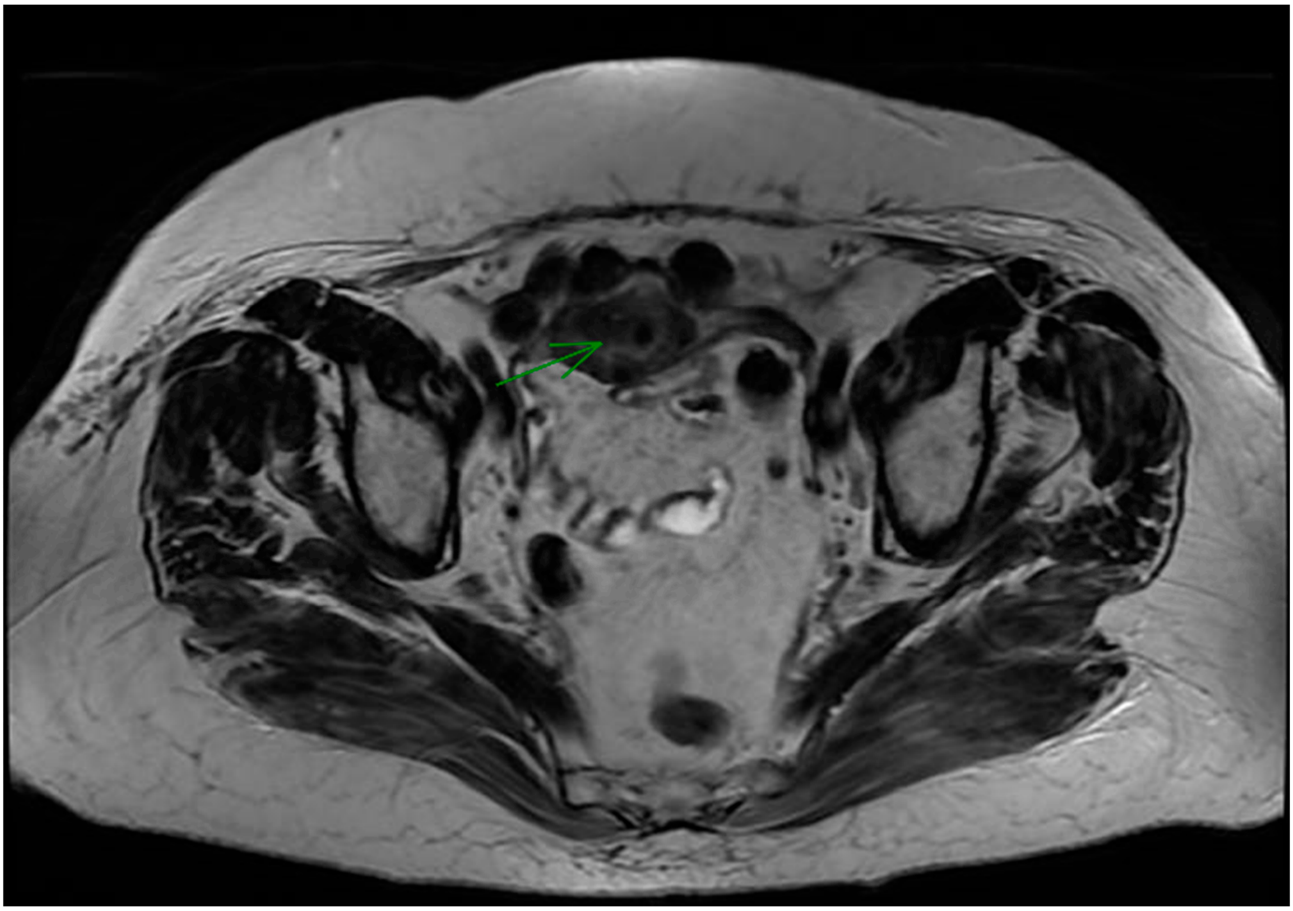

Figure S1. Magnetic Resonance Imaging of the reported case.

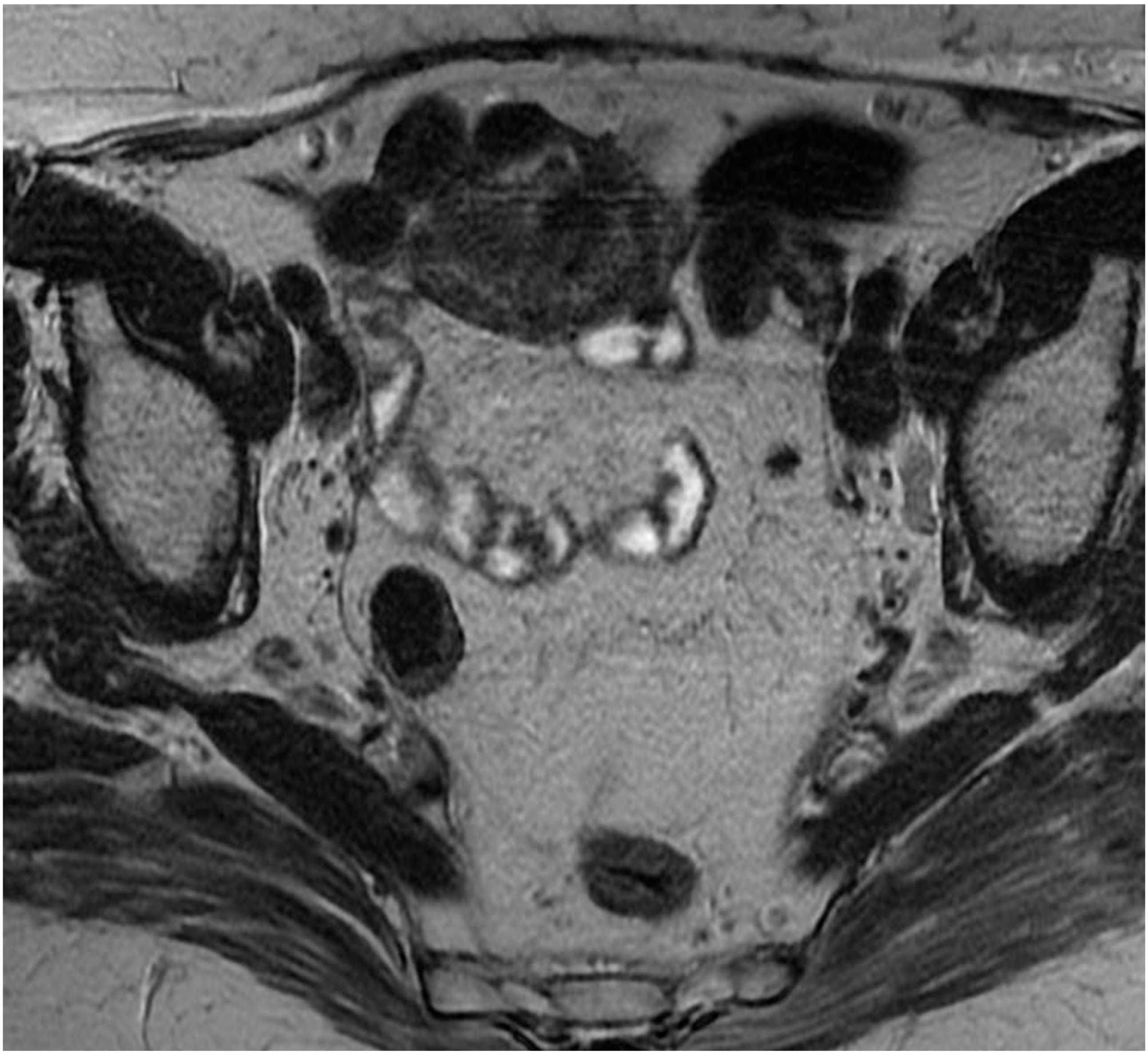

Figure S2. Magnetic Resonance Imaging of the reported case.

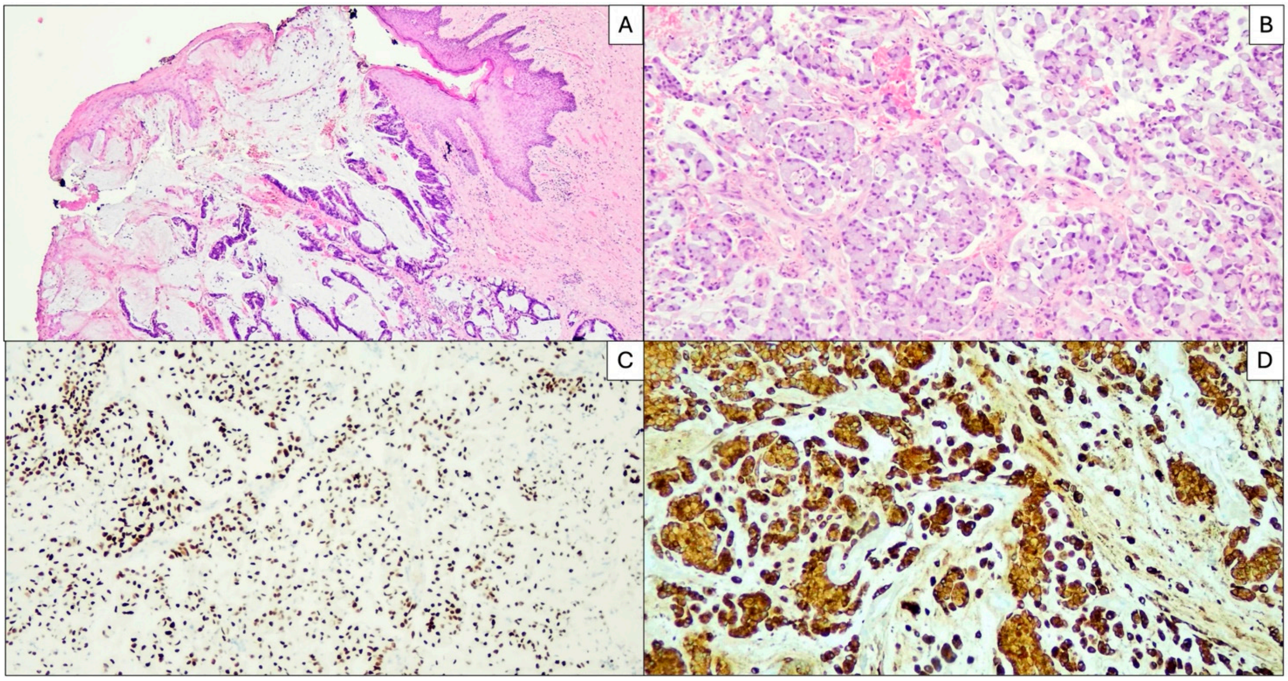

Figure S3. Primary signet ring cell carcinoma of the vulva (A). Low-power view showing an infiltrative mucin-producing adenocarcinoma beneath an overlying squamous epithelium (H&E) (B). High-power view highlighting tumor cells with prominent intracytoplasmic mucin vacuoles displacing the nuclei to the periphery (signet ring morphology) (H&E) (C). Strong and diffuse nuclear positivity for CDX2 and CEA (D).

Supplementary Table S1. Reporting Quality Assessment of Included Case Reports.

Reference (Year)	Patient Characteristics	Condition/Intervention Diagnosis	Description	Outcome Description	Follow-up Included	Diagnostic Tests	Total Score	Notes
Tiltman et al. (1978) [2]	1	1	1	1	1	0	5	Limited information on diagnostic tests.
Kennedy et al. (1993) [3]	1	1	1	1	1	0	5	Limited information on diagnostic tests.
Ghamande et al. (1995) [4]	1	1	1	1	1	1	6	Adequate reporting of patient characteristics, condition/diagnosis, intervention, outcomes, and follow-up. Limited information on diagnostic tests.
Willen et al. (1999) [5]	1	1	1	1	1	1	6	Adequate reporting of patient characteristics, condition/diagnosis, intervention, outcomes, and follow-up. Limited information on diagnostic tests.
Ohno et al. (2001) [6]	1	1	1	1	1	1	6	Adequate reporting of patient characteristics, condition/diagnosis, intervention, outcomes, and follow-up.

								Limited information on diagnostic tests.
Rodriguez et al. (2001) [8]	1	1	1	1	1	1	6	Adequate reporting of patient characteristics, condition/diagnosis, intervention, outcomes, and follow-up. Limited information on diagnostic tests.
Zaidi et al. (2001) [7]	1	1	1	1	1	1	6	Adequate reporting of patient characteristics, condition/diagnosis, intervention, outcomes, and follow-up. Limited information on diagnostic tests.
Liu et al. (2003) [9]	1	1	1	1	1	0	5	Limited information on diagnostic tests.
Dube et al. (2004) [10]	1	1	1	1	1	1	6	Adequate reporting of patient characteristics, condition/diagnosis, intervention, outcomes, and follow-up. Limited information on diagnostic tests.
Dube et al. (2006) [11]	1	1	1	1	1	1	6	Adequate reporting of patient characteristics, condition/diagnosis, intervention, outcomes, and follow-up. Limited information on diagnostic tests.
Cormio et al. (2012) [12]	1	1	1	1	1	1	6	Adequate reporting of patient characteristics, condition/diagnosis, intervention, outcomes, and follow-up. Limited information on diagnostic tests.
Karkouche et al. (2012) [13]	1	1	1	1	1	1	6	Adequate reporting of patient characteristics, condition/diagnosis, intervention, outcomes, and follow-up. Limited information on diagnostic tests.
Musella et al. (2013) [14]	1	1	1	1	1	1	6	Adequate reporting of patient characteristics, condition/diagnosis, intervention, outcomes, and follow-up.

								Limited information on diagnostic tests.
Sui et al. (2016) [15]	1	1	1	1	1	1	6	Adequate reporting of patient characteristics, condition/diagnosis, intervention, outcomes, and follow-up. Limited information on diagnostic tests.
Tulek et al. (2016) [16]	1	1	1	1	1	1	6	Adequate reporting of patient characteristics, condition/diagnosis, intervention, outcomes, and follow-up. Limited information on diagnostic tests.
Matsuzaki et al. (2017) [17]	1	1	1	1	1	1	6	Adequate reporting of patient characteristics, condition/diagnosis, intervention, outcomes, and follow-up. Limited information on diagnostic tests.
He et al. (2017) [18]	1	1	1	1	1	1	6	Adequate reporting of patient characteristics, condition/diagnosis, intervention, outcomes, and follow-up. Limited information on diagnostic tests.
Lee et al. (2017) [19]	1	1	1	1	1	1	6	Adequate reporting of patient characteristics, condition/diagnosis, intervention, outcomes, and follow-up. Limited information on diagnostic tests.
Tepeoglu et al. (2018) [20]	1	1	1	1	1	1	6	Adequate reporting of patient characteristics, condition/diagnosis, intervention, outcomes, and follow-up. Limited information on diagnostic tests.
Kurita et al. (2019) [21]	1	1	1	1	1	1	6	Adequate reporting of patient characteristics, condition/diagnosis, intervention, outcomes, and follow-up. Limited information on diagnostic tests.

Kaltenecker et al. (2019) [22]	1	1	1	1	1	1	6	Adequate reporting of patient characteristics, condition/diagnosis, intervention, outcomes, and follow-up. Limited information on diagnostic tests.
Voltaggio et al. (2019) [23]	1	1	1	1	1	1	6	Limited description of intervention (details of surgery/treatment) and outcomes (complications, long-term results).
Robinson et al. (2020) [24]	1	1	1	1	1	1	6	Adequate reporting of patient characteristics, condition/diagnosis, intervention, outcomes, and follow-up. Limited information on diagnostic tests.
Laforga et al. (2021) [25]	1	1	1	1	1	1	6	Adequate reporting of patient characteristics, condition/diagnosis, intervention, outcomes, and follow-up. Limited information on diagnostic tests.
Martin-Vallejo et al. (2021) [26]	1	1	1	1	1	1	6	Adequate reporting of patient characteristics, condition/diagnosis, intervention, outcomes, and follow-up. Limited information on diagnostic tests.
Moscoso et al. (2021) [27]	1	1	1	1	1	1	6	Adequate reporting of patient characteristics, condition/diagnosis, intervention, outcomes, and follow-up. Limited information on diagnostic tests.
Sato et al. (2022) [28]	1	1	1	1	1	2	7	Adequate reporting of patient characteristics, condition/diagnosis, intervention, outcomes, and follow-up. Detailed description and results for molecular tests.
Sopracorde vole et al. (2023) [29]	1	1	1	1	1	1	6	Adequate reporting of patient characteristics, condition/diagnosis,

								intervention, outcomes, and follow-up. Detailed description and results for molecular tests.
Mateoiu et al. (2024) [30]	1	1	1	1	1	2	7	Adequate reporting of patient characteristics, condition/diagnosis, intervention, outcomes, and follow-up. Detailed description and results for molecular tests.
Natsume et al. (2024) [31]	1	1	1	1	1	1	6	Adequate reporting of patient characteristics, condition/diagnosis, intervention, outcomes, and follow-up. Limited information on diagnostic tests.
Trecourt et al. (2025) [32]	1	1	1	1	1	1	6	Adequate reporting of patient characteristics, condition/diagnosis, intervention, outcomes, and follow-up. Limited information on the description of diagnostic tests.
Fujii et al. (2025) [33]	1	1	1	1	1	2	7	Adequate reporting of patient characteristics, condition/diagnosis, intervention, outcomes, and follow-up. Detailed description and results for molecular tests.
